# Comparative transcriptomics of drought responses in Populus: a meta-analysis of genome-wide expression profiling in mature leaves and root apices across two genotypes

**DOI:** 10.1186/1471-2164-11-630

**Published:** 2010-11-12

**Authors:** David Cohen, Marie-Béatrice Bogeat-Triboulot, Emilie Tisserant, Sandrine Balzergue, Marie-Laure Martin-Magniette, Gaëlle Lelandais, Nathalie Ningre, Jean-Pierre Renou, Jean-Philippe Tamby, Didier Le Thiec, Irène Hummel

**Affiliations:** 1 INRA, Nancy Université, UMR1137 Ecologie et Ecophysiologie Forestières, IFR 110 EFABA, F-54280 Champenoux, France; 2 INRA, Nancy Université, UMR1136 Interactions Arbres Micro-Organismes, IFR 110 EFABA, F-54280 Champenoux, France; 3 Unité de Recherche en Génomique Végétale (URGV), UMR INRA 1165-Université d'Evry Val d'Essonne-ERL CNRS 8196, 2 rue G. Crémieux, CP 5708, F-91057 Evry Cedex, France; 4 UMR 518 AgroParisTech/INRA MIA, 16 rue Claude Bernard, F-75231 Paris Cedex 05, France; 5 Dynamique des Structures et Interactions des Macromolécules Biologiques (DSIMB), INSERM UMR-S 665, Université Paris Diderot-Paris 7, INTS, 6 rue Alexandre Cabanel 75015 Paris, France

## Abstract

**Background:**

Comparative genomics has emerged as a promising means of unravelling the molecular networks underlying complex traits such as drought tolerance. Here we assess the genotype-dependent component of the drought-induced transcriptome response in two poplar genotypes differing in drought tolerance. Drought-induced responses were analysed in leaves and root apices and were compared with available transcriptome data from other *Populus *species.

**Results:**

Using a multi-species designed microarray, a genomic DNA-based selection of probesets provided an unambiguous between-genotype comparison. Analyses of functional group enrichment enabled the extraction of processes physiologically relevant to drought response. The drought-driven changes in gene expression occurring in root apices were consistent across treatments and genotypes. For mature leaves, the transcriptome response varied weakly but in accordance with the duration of water deficit. A differential clustering algorithm revealed similar and divergent gene co-expression patterns among the two genotypes. Since moderate stress levels induced similar physiological responses in both genotypes, the genotype-dependent transcriptional responses could be considered as intrinsic divergences in genome functioning. Our meta-analysis detected several candidate genes and processes that are differentially regulated in root and leaf, potentially under developmental control, and preferentially involved in early and long-term responses to drought.

**Conclusions:**

In poplar, the well-known drought-induced activation of sensing and signalling cascades was specific to the early response in leaves but was found to be general in root apices. Comparing our results to what is known in arabidopsis, we found that transcriptional remodelling included signalling and a response to energy deficit in roots in parallel with transcriptional indices of hampered assimilation in leaves, particularly in the drought-sensitive poplar genotype.

## Background

Water deficit is recognised as one of the main environmental constraints restricting natural and agro-ecosystem productivity [1,2]. The influence of water availability on plant productivity suggests that water limitation has shaped the natural variation and evolution of many physiological traits [3]. Biotechnology has investigated the genetic basis of drought tolerance by targeting relevant genes [4,5]. However, manipulating a single gene at a time, even genes encoding transcription factors, has proved insufficient to maintain productivity under drought; the tendency of cell systems is to restore homeostasis and mutants showing improved drought-or salt-tolerance are often stunted [6,7]. Transcriptome meta-analyses and regulon detection could contribute to identifying physiological processes relevant to drought response. The availability of genomic tools such as high density arrays or whole-genome microarrays has led to an increasing number of studies examining drought-induced transcriptional remodelling [8-15].

Drought tolerance, which is obviously a multigenic trait, clearly depends on genome x environment interactions [16]. Comparative genomics has emerged as a promising means of unravelling the molecular networks underlying complex traits [17,18]. Studies analysing gene expression in parallel with quantitative trait loci (QTL) have shown that drought-induced changes in gene expression in specific genotypes were consistent with the observed physiological responses [11,19]. Wilkins *et al*. highlighted the genotype specificity of the transcriptome response occurring in poplar leaves under drought conditions [14]. Two drought-tolerant genotypes of maize showed more rapid and drastic changes in gene expression under drought, especially during recovery, than a drought-sensitive genotype [13]. Moreover, drought tolerance relies on physiological adjustments occurring in distinct organs, involves the interplay of signalling/sensing cascades, and supposes integrated responses from molecular to whole-plant level. The few studies that have compared leaf and root transcriptomes have highlighted the organ specificity of drought responses [9,20,21]. Roots sense the edaphic water deficit, send chemical signals to shoots, and maintenance of root growth despite reduced water availability can contribute to drought tolerance through water foraging [22]. Species-dependent features also shape the transcriptome response; almost none of the 27 genes reliably responsive to water stress in arabidopsis were regulated under drought in poplar and pine [7,23]. Besides, many genes "inducing drought tolerance" have been identified under an abrupt and/or severe stress, which is a far cry from a realistic, slow developing and long-lasting drought [8,24]. By quantifying and controlling water deficit by physical variables such as soil relative water content, the two genotypes experienced a similar degree of water deficit, allowing a robust comparison of their physiological and molecular responses [25]. Such an ecophysiological approach has proved to be an efficient means of comparing drought response across genotypes [26,27].

Since the publication of the *Populus trichocarpa *genome in 2006, poplar has become the model species for trees and the most studied tree species overall [28]. In addition, poplars are also ecologically and economically important. Poplar inter-specific hybrids (*Populus *spp.) are among the fastest-growing trees under temperate latitudes and are grown for pulp, paper wood and fuel production purposes [29]. While poplars are known to be sensitive to water deprivation as compared to other trees, drought tolerance varies considerably between genotypes of *Populus*, both inter-and intra-specifically [30,31]. The purpose of the present study was to analyse the transcriptome responses induced by mild-to-moderate water deficit in two poplar genotypes. On the one hand, we applied a short-term water deficit to access sensing and signalling events; on the other, we imposed prolonged water deficit to reveal the molecular controls of plant performance under steady state stress. Transcriptome responses were analysed in mature leaves and in growing root apices in order to gain a wide assessment of the response and to draw an integrative picture of molecular responses to drought. The comparison of two genotypes known to differ in their drought tolerance revealed not only reliable drought markers but also the divergences and similarities in transcriptional networks, highlighting candidate genes for future diversity screening.

## Results

### Experimental Design

We focused on two hybrid genotypes ('Carpaccio' and 'Soligo') exhibiting contrasting tolerance to drought in field experiments, Carpaccio productivity being less hampered by drought than that of Soligo [32]. Young trees were submitted either to a short-term water deficit by withholding irrigation 36 hours before harvest [early response (EAR)] or to a 10 day-long treatment, inducing a mild drought [long-term response to mild stress (LMI)] or a moderate drought [long-term response to moderate stress (LMO)].

**Figure 1:**
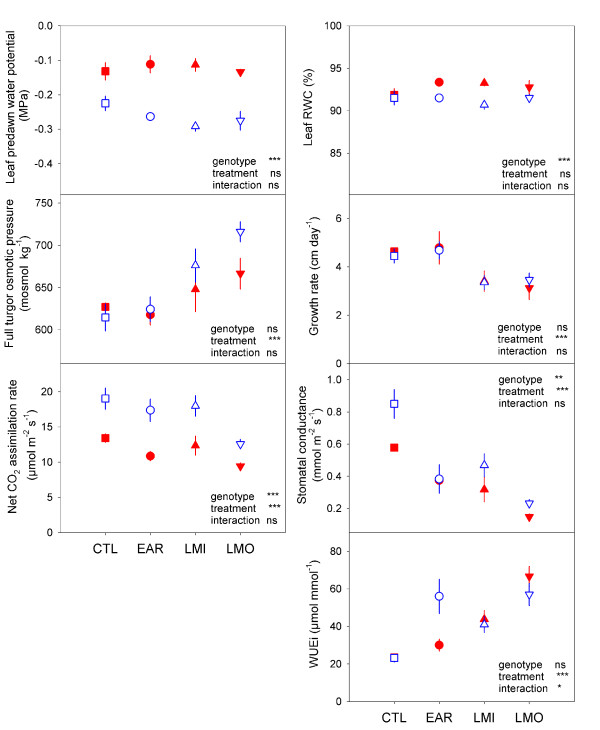
**Ecophysiological responses**. Leaf predawn water potential, leaf relative water content, leaf full turgor osmotic pressure, height growth rate, net CO_2 _assimilation rate, stomatal conductance and instantaneous water use efficiency (WUE_i_) were measured on a dedicated batch of trees at the harvest time point. Closed red symbols: Carpaccio, open blue symbols: Soligo; CTL, EAR, LMI, LMO: treatments. Mean ± s.e., n = 6.

None of the water deficit treatments modified leaf water status. However leaf predawn water potential and leaf relative water content differed significantly between genotypes (Figure 1). Leaf full turgor osmotic pressure increased in response to long-term stresses, indicating active osmotic adjustment, especially in Soligo. In both genotypes, the EAR treatment was too brief to affect either leaf full turgor osmotic pressure or stem growth rate. Long-term stresses reduced growth in height similarly in both genotypes. Under all treatments, including controls, gas exchange rates were significantly higher in Soligo. LMO reduced the net CO_2 _assimilation rate by 30% in both genotypes. Stomatal conductance was reduced under all drought treatments-more strongly in response to LMO than in EAR and LMI, reflecting the applied stress level. The sharp drop in stomatal conductance was responsible for the higher instantaneous water use efficiency (WUE_i_) in Soligo under EAR treatment. In long-term treatments, WUE_i _was enhanced similarly in Carpaccio and Soligo. While contrasting drought tolerance has been assessed in the field, the physiological adjustments diverged only weakly between genotypes under our mild-to-moderate water deficit experiments carried out on juvenile trees in the greenhouse. The ecophysiological responses observed were consistent with the level and duration of each treatment, indicating that trees sensed and responded to the water deficits applied.

An Affymetrix poplar genome array was used to assess genome-wide expression in mature leaves and root apices. The array, which contains 61,251 probesets representing over 56,000 transcripts and predicted genes, was generated from several *Populus *species. As we were dealing with a comparative approach, we checked the suitability of this array for both genotypes by hybridizing with genomic DNA. This point is important as transcript sequences might have diverged in the two genomes, which could lead to absence of hybridization without expression meaning [33]. Genomic arrays were screened in parallel with expression arrays to extract probesets informative for both genotypes. Analysis of signal intensity highlighted that 20% of probesets never matched and 18% of probesets were detected on only genomic DNA arrays, *i.e*. these genes were never transcribed under our conditions (Table 1). Within this class, 768 probesets were Carpaccio-specific and 518 Soligo-specific, indicating a similar level of divergence in the two genomic sequences. Based on gene expression patterns, we also filtered out the 346 and 620 probesets that hybridized exclusively to Soligo or Carpaccio sequences, respectively. Indeed, the absence of signal in all arrays dedicated to one of the two genotypes, including genomic arrays, was interpreted as a genotype-specific modification of the target sequence leading to a technical mismatch, rather than a between-genotype difference in expression. Further analyses were performed on the remaining 36,687 probesets, which clearly matched both Carpaccio and Soligo. Targeted genes were expressed mostly in both organs while most genotype-specific sequences were expressed preferentially in a single organ.

**Table 1:** Genomic DNA-based selection of probesets

	Exclusive assignation	Number of probesets	
Both genotypes	No signal	12,530	
	Present only on genomic DNA arrays	9,782	

1-Genotype-specific hybridization			

Present only on Carpaccio arrays	Present only on genomic DNA arrays	768	
	
	Expressed in root and leaf	154	
	Root-preferred expression^1^	280	620
	Leaf-preferred expression^1^	186	

Present only on Soligo arrays	Present only on genomic DNA arrays	518	
	
	Expressed in root and leaf	15	
	Root-preferred expression^1^	140	346
	Leaf-preferred expression^1^	191	

2-Probesets matching on both genotype arrays			

Both genotypes expression arrays	Expressed in root and leaf	26,834	
	Root-preferred expression^1^	4,573	
	Leaf-preferred expression^1^	3,693	

Both genotypes genomic DNA arrays	Only on Carpaccio expression arrays		651
	Only on Soligo expression arrays		936

	Sum		61,251

^1^Gene expression patterns were assessed without regards to growth conditions, i.e. if transcripts are present in the two replicates of at least one of the 4 conditions dedicated to the organ (root apices or mature leaves) per genotype.

### Transcriptome response in root apices is consistent across treatments and genotypes but contrasted in leaves

To assess drought-driven transcriptome responses, the expression data of drought-treated trees were compared with their respective controls within organs and genotypes. Pair-wise correlation provides a global view of the changes in gene expression across conditions (Table 2). Pearson coefficient values were low, in some cases non-significant, between leaf and root transcriptomes, indicating organ-contrasting transcriptome responses to drought. Drought-driven changes in gene expression occurring in root apices were strongly consistent, regardless of the conditions compared. In mature leaves under prolonged drought, a genotype specificity can be suspected, as correlations between LMI and LMO responses within each genotype were stronger than all other between-genotype comparisons. In contrast, considering EAR treatment, the best correlation was detected between genotypes, showing that early responses could be distinguished from long-term responses. This global overview showed that between-genotype differences were exacerbated in leaves given the consistency of root transcriptome responses. Our experimental design did not deal with pure dose-or time-dependant drought treatments. However co-variations between changes in gene expression in all three conditions were detected, indicating similarities in the transcriptome responses.

**Table 2:** Correlation between changes in gene expression in the twelve conditions

		CL	CL	CL	SL	SL	SL	CR	CR	CR	SR	SR	SR
		
		EAR vs CTL	LMI vs CTL	LMO vs CTL	EAR vs CTL	LMI vs CTL	LMO vs CTL	EAR vs CTL	LMI vs CTL	LMO vs CTL	EAR vs CTL	LMI vs CTL	LMO vs CTL
**CL**	**EAR vs CTL**												
**CL**	**LMI vs CTL**	0.381											
**CL**	**LMO vs CTL**	0.204	0.613										
**SL**	**EAR vs CTL**	0.430	-0.03	-0.14									
**SL**	**LMI vs CTL**	*0.015 ns*	0.078	0.191	0.150								
**SL**	**LMO vs CTL**	0.142	0.284	0.397	0.245	0.583							
												
**CR**	**EAR vs CTL**	0.113	*-0.01 ns*	*-0.01 ns*	0.064	0.146	0.101						
**CR**	**LMI vs CTL**	0.163	0.157	0.072	0.073	0.147	0.150	0.663					
**CR**	**LMO vs CTL**	0.159	0.077	0.023	0.080	0.073	0.092	0.650	0.775				
**SR**	**EAR vs CTL**	0.040	-0.06	-0.02	0.066	0.166	0.109	0.756	0.443	0.323			
**SR**	**LMI vs CTL**	0.176	0.172	*0.005 ns*	0.084	0.063	0.080	0.542	0.610	0.668	0.518		
**SR**	**LMO vs CTL**	0.149	0.049	0.025	0.129	0.130	0.150	0.700	0.639	0.581	0.779	0.746	

Pearson coefficient values between relative expression data (C: Carpaccio, S: Soligo; L: mature leaves, R: root apices; CTL, EAR, LMI, LMO: treatments). All given values are significant (*P *< 0.0001; N = 36,687 probesets) unless specified otherwise by ns (non significant).

**Figure 2:**
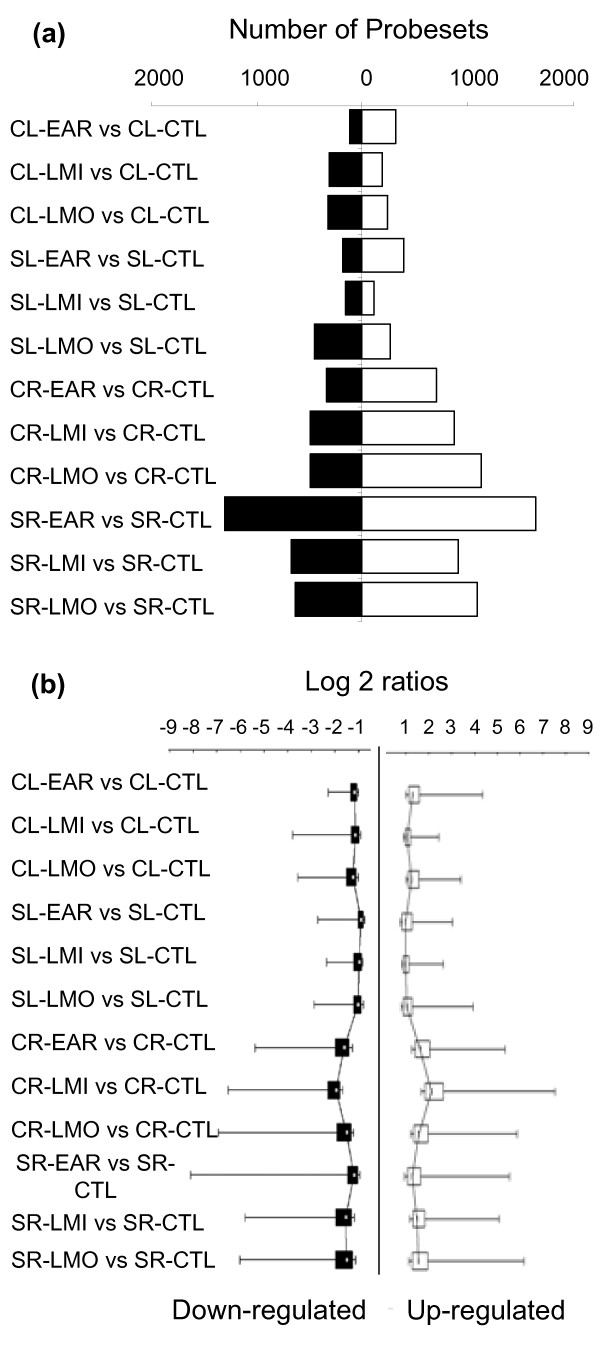
**Analysis of significant drought-driven regulation**. A total of 6,725 probesets exhibited at least one significant difference in normalized signal intensity between treated and respective control arrays (t-test, Bonferroni *P *< 0.05). (a) Number of probesets corresponding to regulated genes in response to each condition. (b) Intensities of drought-driven regulation in each condition. Log 2 ratio distributions are shown by box-and-whisker plots. The central mark is the median, the edges are the 25th and 75th percentiles, and the whiskers extend to minimum and maximum values. Up-regulation is depicted in white and down-regulation in black.

One-fifth of the 36,687 probesets displayed a significant change in signal intensity in response to drought in at least one pair comparison (6,725 probesets, Additional file 1), either in roots or in leaves. As expected from the experimental design, which considered several organs, genotypes and treatments simultaneously, more than half of the significant drought-driven regulations occurred only once across the 12 comparisons. Drought-driven regulations were distributed unequally among the 12 comparisons (Figure 2a). The leaf transcriptome appeared less drought-responsive than the transcriptome of root apices (2,120 *versus *5,331 significantly affected probesets, respectively), which might reflect, in part, the higher sensitivity of an actively growing tissue to water deprivation. EAR-treated Soligo roots exhibited more drought-driven regulation than the other conditions although rather weak in intensity, consistent with the lower variance of the comparison (Figure 2). In contrast in Carpaccio, drought yielded stronger regulation in terms of median or extreme values (Figure 2b). When carried out on all regulated genes, quantitative analysis revealed the high responsiveness of the root transcriptome and confirmed the existence of genotype specificity of transcriptome responses.

### Functional categories involved in drought-induced transcriptome responses

Drought-responsive probesets were assigned to poplar gene models and annotated using web-based queries. Screening allowed the annotation of almost all probesets. Only 89 out of the 6,725 probesets were neither assigned to a poplar gene model nor functionally annotated. For the sake of brevity, and given the assumption that best homology implies a true ortholog, gene names of the closest arabidopsis genes are used to describe poplar genes. An overview of functional groups involved in drought responses was obtained through singular enrichment analysis (SEA, Additional file 2). Concerning the Cellular Component ontology, "endomembrane system" (GO:0012505) and "cell wall" (GO:0005618) were sorted in response to all drought conditions and were highly enriched for Soligo roots (EAR down-regulation). Another term, "nucleus" (GO:0005634), was enriched in the up-regulated responses to EAR for both organs and to all drought conditions for Soligo roots, whereas it was found among the down-regulated responses for LMO-treated Carpaccio roots. The most enriched terms of Biological Processes ontology were "response to stimulus" (GO:0006950), "response to abiotic stimulus" (GO:0009628), "response to stress" (GO:0006950) and "response to endogenous stimulus" (GO:0009719). These enrichments were seen for both organs under all conditions and indicated not only that the constraint was perceived--although the treatments were only mild-to-moderate--but also that prolonged drought remained a stressful growing condition. Interestingly, "growth" (GO:0040007) was specifically enriched for Soligo roots (EAR down-regulation). For mature leaves, metabolisms responded differentially in the two genotypes. "Primary metabolic process" (GO:0044238) was enriched for Carpaccio (LMI up-regulation). In contrast, for Soligo, "photosynthesis" (GO:0015979) and "secondary metabolic process" (GO:0019748) were detected among the down-regulations occurring in response to short and prolonged drought, respectively.

In terms of Molecular Function ontology, the most significant enriched term was "binding" (GO:0005488). Consistent with this, "transcription factor activity" (GO:0003700) and "transcription regulation activity" (GO:0030528) were sorted under all treatments (up and down regulations). "Transporter activity" (GO:000521) was enriched for Soligo leaves (LMI up and down regulations) and for Carpaccio roots (LMI and LMO up-regulations). Other GO terms, such as "catalytic activity" (GO:0003824), "hydrolase activity" (GO:0016787), and "transferase activity" (GO:0016740) were found in most lists.

In order to detect physiologically relevant patterns, groups of functionally related genes were identified using iterative group analysis (iGA) [34]. Functional groups of genes were delineated using not only GO terms but also any keywords from gene annotations. The iGA procedure revealed concerted changes in functional groups (Table 3, see Additional file 3 for details). Concerning leaves, in Carpaccio, "*ABI5 binding protein*" and "*9-cis-epoxycarotenoid dioxygenase" (NCED) *were significantly up-regulated and "*pyarabactin resistance-like" (PYL) *significantly down-regulated in response to EAR, indicating an involvement of ABA biosynthesis/signalling pathways. In contrast, in Soligo, EAR induced a generic response to stress, including regulations of signal transduction, transcription and metabolic processes, while the ABA signalling/response was detected in response to LMO (up-regulated: "*protein phosphatase type-2C" (PP2C)*, "*RARE-COLD-INDUCIBLE" (RCI2A)*, "*XERICO*"). Similarly, "*galactinol synthase*" was up-regulated in response to EAR in Carpaccio and by prolonged drought in Soligo. In Carpaccio subjected to prolonged drought, up-regulated groups were related either to cell redox homeostasis ("*superoxide dismutase*", "*metallothionein*") or to cell rescue processes ("*RCI2A*"), and included an LMI-specific "*DEAD-box RNA helicase*". Soligo responses to prolonged drought were characterized by down-regulated "*transporter*".

**Table 3:** Drought-dependent enrichment of functional groups in leaf arrays

Up-regulated groups	PC	%	Down-regulated groups	PC	%
**CL-EAR vs CL-CTL**					

Major intrinsic protein	3.7E-05	60	Unknown protein/DUF247	6.1E-04	100
Unknown protein/ABI5 binding	2.0E-03	100	Bet v I allergen/PYL	1.5E-03	100
Galactinol synthase-like	6.3E-03	50			
Chitinase activity	6.4E-03	100			
Carotene dioxygenase activity/NCED	9.7E-03	100			

**CL-LMI vs CL-CTL**					

Metal ion binding/SOD, metallothionein	1.7E-03	67	β-glucosidase activity	2.1E-06	100
ATP dependant helicase/DEAD-box	2.6E-03	100			
RNA binding	4.8E-03	100			
Unknown protein/RCI2A	6.3E-03	100			

**CL-LMO vs CL-CTL**					

Catalyticactivity/Esterase/lipase/thioesterase	1.3E-05	78	Leucine-rich repeat	3.2E-04	70
Nutrient reservoir activity/Germin, Extensin-like	1.5E-04	83	Protein amino-acid phosphorylation/Protein kinase	1.6E-03	100
Flavonoid 3'-monooxygenase activity	2.3E-03	100	Calcium ion binding/EF-hand	3.8E-03	100
Cell redox homeostasis/Glutaredoxin	4.6E-03	50			
Metal ion binding/SOD, metallothionein	8.0E-03	60			
Zinc ion binding	8.2E-03	27			
DNA binding	9.2E-03	24			
Unknown protein/RCI2A	9.3E-03	67			

**SL-EAR vs SL-CTL**					

Calcium ion binding/EF-hand	2.6E-06	89	B-glucosidase activity	8.2E0-8	100
DNA binding/WRKY	3.0E-04	59	Regulation of transcription, DNA-dependent	2.3E-03	19
Protein amino acid phosphorylation	1.1E-03	100	Unknown protein/DUF247	2.5E-03	100
ATP binding	9.7E-04	98	Photosynthesis	5.4E-03	67
Ankyrin repeat family protein	9.5E-03	100	ATP synthesis coupled proton transport	6.7E-03	100
			ATP binding	9.3E-03	100
			Protein binding	9.0E-03	43
			RNA binding	9.0E-03	100

**SL-LMI vs SL-CTL**					

Galactinol synthase-like	9.0E-03	50	Tetratricopeptide repeat-protein	4.0E-04	40
			Membrane	5.3E-03	100
			Cysteine-type peptidase activity/Papain	6.2E-03	100
			UDP-glucosyltransferase	7.3E-03	100
			Drug transporter activity/MatE	7.4E-03	75

**SL-LMO vs SL-CTL**					

Protein ser/thr phosphatase activity/PP2C	1.1E-04	100	O-glucosyl hydrolase activity/β-glucosidase	2.9E-04	60
Unknown protein/RCI2A	3.8E-04	100	Tetratricopeptide repeat-protein	4.1E-04	50
ABA metabolic process/Xerico	2.4E-03	100	Amino acid transport	8.8E-03	100
Galactinol synthase-like	5.6E-03	33			
No apical meristem (NAM) protein	5.5E-03	100			
Two-component signal transduction	8.5E-03	100			

Groups of functionally related genes were identified by iGA. The probability of change (PC) and the number of changed versus total group numbers (%) are given. Significantly regulated groups are shown (PC-value < 0.01).

In roots (Additional file 3), many functional groups were detected in accordance with the strong responsiveness to drought of the root transcriptome. In these large lists, the iGA procedure revealed a striking conservation of the concerted changes in most conditions, highlighting a common response to drought. Whatever the condition, we detected enrichment in groups of genes known to be responsive to abiotic stress and/or drought. The generic response involved genes that were related to i) ABA biosynthesis/signalling (up-regulated: "*NCED*", "*PP2C*"; down-regulated: "*PYL*"), ii) cell rescue and/or cell redox homeostasis (up-regulated: "*dnaJ*", "*heat shock protein*", "*glutathion-S-transferase*", "*metallothionein*"), and iii) the response to hypoxia (down-regulated: "*alcohol dehydrogenase*"; "*pyruvate decarboxylase*", "*LOB domain-containing protein*"). As expected for actively growing organs, stress impacted recurrent groups of genes that were involved either in expansion (up-regulated: "*aquaporins*"; down-regulated: "*pectinesterase*", "*L-ascorbate oxidase*") or in meristematic activity and cell cycle (down-regulated: "chromosome organization", "DNA replication"). EAR-treated Soligo roots underwent an extensive metabolism-related response (up-regulated: "*raffinose synthase*", "*asparagine synthase*", "*trehalose phosphatase*"; down-regulated: "nucleotide-sugar metabolism", "fatty acid desaturation"). In addition, an erosion of the "transcription factors" group was detected across treatments. In Carpaccio roots, a "transcription factors" group of nine up-regulated genes was detected in response to EAR (putative "*Homeobox proteins" (ATHB12*, *ATHB40*, *ATHB6)*, "*Responsive to desiccation" (RD26)*, "*Nuclear transcription factor Y, alpha" (NF-YA*), "*Heat shock transcription factor C1" (AtHSFC1)*, "*ABA-Repressor1" (ABR1)*), whereas five up-regulated genes were detected under LMI (putative "*ATHB12*", "*ATHB40*", "*WRKY*"), and only one under LMO. Concerning Soligo, we detected five "transcription factors" groups in response to EAR (38 genes including putative "*ATHB12*", "*RD26*", "*ABR1*", "*AtHSFC1*"), two groups under LMI (3 genes including putative "*ATHB12*", "*RD26*") and no enrichment under LMO. As highlighted by this functional categorization, the applied treatments clearly drove transcriptome responses in both organs and genotypes. Both procedures provided consistent results, enabling the extraction of processes physiologically relevant to drought responses. Several genes of interest can be discriminated on the basis of their contribution to enriched functional categories.

### Analysis of drought-responsive gene networks based on gene co-expression relationships provides robust drought markers and candidate genes

**Figure 3:**
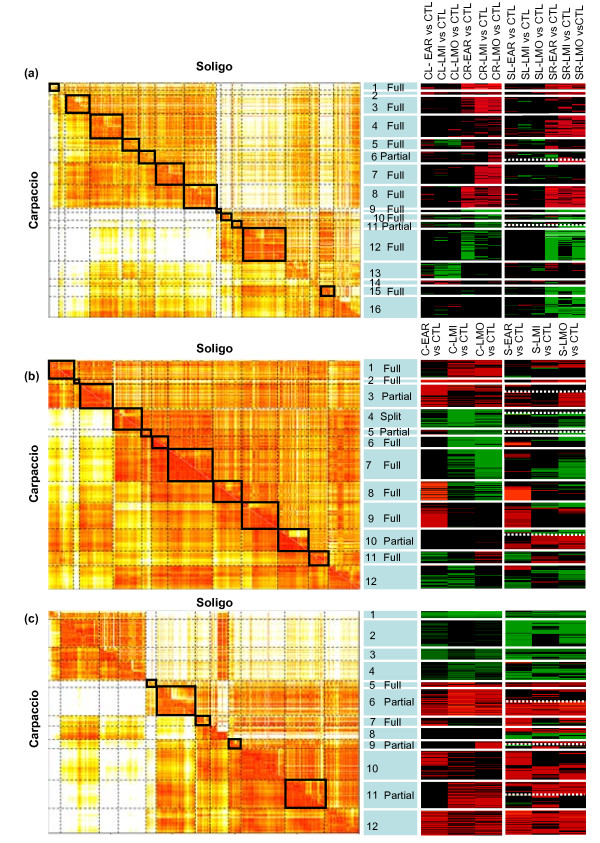
**Global comparison of Carpaccio and Soligo drought responses based on differential clustering analysis**. DCA were performed (a) on the 3,515 probesets that were significantly regulated at least twice across the twelve conditions (t-test, Bonferroni *P *< 0.05); (b) on the 652 probesets that were significantly regulated in mature leaves, at least twice across the six combinations; and (c) on the 2,410 probesets that were significantly regulated in root apices at least twice across the six combinations. Complete distance matrices were combined into a single matrix (left panel with small distance in red and large distance in white). Full, partial or split conservation were given in the middle panel (blank = not conserved). Expression profiles are shown for Carpaccio and for Soligo (right panel with significant up-and down-regulation indicated in red and green, respectively).

Conservation of co-expression patterns between the two genotypes was investigated using a differential clustering algorithm (DCA) [35,36]. This approach is a two-step procedure that (*i*) defines transcriptional groups of co-expressed genes in one genotype (referred to as the "reference" genotype), and (*ii*) evaluates, for each transcriptional group defined in step 1, its level of conservation in the other genotype (referred to as the "target" genotype). In this study, we chose Carpaccio as the reference genotype and Soligo as the target genotype (Figure 3). To avoid chance associations, the DCA procedure was carried out on subsets of genes that were significantly regulated at least twice across all conditions (thus taking into account about half of the drought-driven regulatory patterns). Co-expression relationships between genes were assessed on the basis of expression modifications occurring across drought conditions, either in the two organs (Figure 3a), or separately in mature leaves (Figure 3b) and root apices (Figure 3c). The reliability of the procedure was highlighted by the agglomeration of probesets that targeted identical gene models in consistent modules (Additional file 4).

In a joint analysis of drought-driven regulation in leaves and roots, 16 clusters of co-expressed genes were first identified in Carpaccio (Figure 3a). The DCA procedure revealed 10 transcriptional modules as fully conserved between the two genotypes (clusters 1, 3, 4, 5, 7, 8, 9, 10, 12 and 15). Delineation of these transcriptional groups confirmed the contrasting gene regulation in mature leaves and growing root apices. Four clusters (13-16) appeared to be relatively distant from each other compared to two homogeneous sets of clusters (clusters 1-8, 1341 up-regulated genes; clusters 9-12, 565 down-regulated genes). In between, some co-regulation relationships appeared to be partially conserved (clusters 6 and cluster 11), meaning that a subset of the genes that are co-expressed in the reference genotype lost their co-expression relationships in the target genotype. This phenomenon applied to 39 genes (with transporters of different substrates included in cluster 6, subset b, see Additional file 4) and 28 genes (with transcription factors and hormone biosynthetic enzymes, such as putative *Allene oxide synthase *included in cluster 11, subset b, see Additional file 4). Finally, the large divergence of co-expression relationships between genes in Carpaccio and Soligo were highlighted (clusters 2, 13, 14 and 16, totalling about 600 genes). Up-regulated cluster 2 included several *Aquaporins *and genes related to ABA signalling or the response to oxidative stress (Additional file 4). Loss of co-expression relationships between genes was due mainly to almost invariant gene expression in Carpaccio (cluster 16) or in Soligo (clusters 13 and 14). These two latter transcriptional modules collected genes with a similar annotation (*Unknown proteins*, putative *Cytochrome P450*, *Leucin-rich repeat proteins*) or associated with "cell wall" or "transport activity" (putative *Wall-associated kinases*, *Amino-acid permeases*).

In mature leaves, drought-driven regulation of gene expression was weak (Figure 2) and led to the clear distinction of 12 transcriptional modules (Figure 3b). Drought-driven transcriptome responses appeared strongly dependent on stress duration, splitting clearly into early and long-term responses in accordance with global gene expression analyses (Table 2). Most transcriptional modules of co-expressed genes were conserved. On the one hand, some conserved modules were regulated exclusively in response to prolonged (clusters 1, 6 and 7) or short (cluster 9) drought. On the other hand, conserved clusters 8 and 11 encompassed 81 genes that were responsive to all drought conditions but that were inversely regulated in short and in prolonged drought. The contrast between short and prolonged drought responses in mature leaves relied on the regulation of 301 distinct genes (Additional file 4). The DCA procedure also revealed drought marker genes that were up-regulated in both genotypes and under all drought conditions (conserved cluster 2; including putative *RCI2A*, *ATHB12*, *Galactinol synthase AtGolS2*, *ABC transporter*). The sole transcriptional module identified as not-conserved between the two genotypes (cluster 12) resulted partly from the genotype-specific response to LMI (which repressed a lower number of genes in Soligo than in Carpaccio, Figure 2a). Far more informative were the partially conserved clusters (clusters 3, 5, and clusters 4, 10, Additional file 4). Genes were almost invariant in Soligo but strongly regulated in Carpaccio in sub-group 3b (24 up-regulated genes, e.g. putative *Aquaporin*, *Flavonol synthase*, five transcription factors) and in sub-group 5b (nine down-regulated genes including three putative *Wall-associated kinases*). In cluster 4b, genes were strongly repressed by LMI in Carpaccio leaves but were not drought-responsive in Soligo (15 genes including putative *β-xylosidase1*, *Pectinesterase*, 5 genes related to defence response). Conversely, genes that were almost invariant in Carpaccio and drought-responsive in Soligo were split into up-regulated sub-group 10a (including hormone signalling of jasmonic acid, auxin and ABA) and down-regulated sub-group 10b (including putative *ATP sulfurylase*, *Squalene epoxidase*, *Gibberellin-2-β-dioxygenase2*).

In root apices, the strong intensity of drought-driven expression patterns allowed 12 transcriptional modules to be defined unambiguously (Figure 3c, left panel). Two distant sets of clusters could be discriminated (down-regulated clusters 1-4, totalling 612 genes, and up-regulated clusters 5-12, totalling about 1300 genes). The down-regulated set included genes related to cell cycle and DNA processes, notably putative *cyclin-dependent protein kinases *and *DEAD-box RNA helicases*. The up-regulated groups included genes related to metabolic processes (putative *Phosphoenolpyruvate carboxylase*; *Dihydrodipicolinate reductase1*, *Alternative oxidase*), and to catabolism (putative *Ubiquitin-protein ligases*). As shown in Figure 3c, Carpaccio transcriptional modules were delineated according to differences in intensity of gene regulation across treatments. This clustering outline was valid for both genotypes, which is consistent with the high correlation observed between treatments and genotypes (Table 2). However, most of the transcriptional modules were labelled "not conserved". In these cases, the homogeneous response, in terms of up or down regulation, masked the fine genotype-specific tuning of transcriptome responses that was revealed by the DCA procedure. In cluster 8 only, the expression pattern differed widely between genotypes. Besides, some modules were labelled "fully" or "partially" conserved, indicating that genes conserved their co-expression properties (clusters 5 and 7, sub-groups 6a, 9a and 11a, totalling about 472 genes, Additional file 4). In root apices, drought-driven regulation was highly consistent in both genotypes, and it was the differential tuning across drought conditions that accounted specifically for the loss of conservation in gene co-expression relationships.

### Common and specific components of drought-induced response in different organs

**Figure 4:**
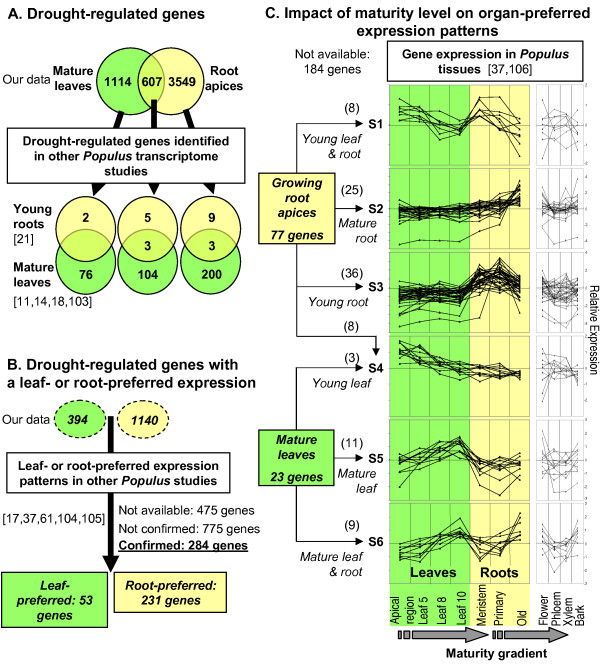
**Expression and regulation of drought-responsive genes in other Populus species**. A. Detection of drought-regulated genes common to our study and the literature. B. Detection of organ-preferred expression through comparison of our data with the literature. C. Detection of the impact of maturity level on gene expression in interaction with organ specificity using exPlot [37]. Meta-analysis was restricted to the literature considering root and/or leaf tissues.

In order to test the consistency of drought-regulated genes across species and organs, we cross-referenced our results with the poplar literature regardless of pattern of regulation (Figure 4). Of the 5270 drought-regulated genes found here, 402 had already been identified as drought-responsive in leaves or roots of other *Populus *species. Among them, *XERICO*, *RD26*, *ATHB12*, *Arabidopsis thaliana drought-induced 21(ATDI21)*, *Metallothionein 2A *(*MT2A*) and *Metallothionein 3 *(*MT3) *were confirmed as robust drought markers as they were drought-responsive in most studies. Our analysis also highlights the finding that regulation previously detected in leaves also occurs in root apices and vice versa (Figure 4A, Additional file 5). The low conservation level of gene lists across studies might arise from differences in genotypes, organs, drought treatments, molecular platforms and statistical analyses.

Our experimental design did not allow unequivocal testing of organ-preferred expression since tissue specificity (root versus leaf) and maturity level (growing versus mature) were confounded. To our knowledge, only one study has compared leaf and root transcriptomes under drought, thus precluding a robust meta-analysis of the differential impact of drought according to the organ. Given that gene expression patterns between leaf and root have been compared more extensively under optimal conditions, we delineated two subsets of drought-regulated genes on the basis of consistent expression pattern in control and drought treatment (Figure 4B). Among drought-regulated genes with a leaf-preferred expression pattern, one-fourth was confirmed to be expressed preferentially in leaf in other *Populus *species. A similar result was found for genes expressed preferentially in root. The expression patterns of 775 genes were not confirmed in other *Populus *species and the patterns of 475 genes could not be tested due to missing information. To our knowledge, the interaction of organ maturity and drought on the transcriptome responses has not been documented in poplar. Although maturity level could affect the sensitivity of the transcriptome response to drought, we searched for expression profiles in young and mature organs under optimal conditions in another *Populus *species (exPlot) [37]. From the two subsets of drought-regulated genes in our study, we extracted six contrasting expression profiles under optimal conditions (Figure 4C). S1 gathers potential markers of growing tissues and could also be drought-regulated in growing leaves. In contrast, genes in S2 and S3 were confirmed as being expressed preferentially in root, either in old roots (S2) or in young roots (S3). Genes that are expressed preferentially in young leaves under optimal conditions were drought-regulated in growing roots or mature leaves (S4). Among genes expressed preferentially in mature leaves in our experiment, 20 were confirmed to be expressed preferentially in mature tissue, either in leaf (S5) or in both leaf and root (S6). Although indirect (i.e. extrapolated from other *Populus *species grown under optimal conditions), these arguments strengthen the hypothesis of an organ-preferred expression of some drought-regulated genes. However, missing information precludes a robust meta-analysis allowing the drought responses within the *Populus *genus to be unravelled. Given that most drought transcriptome studies have focused on mature leaves, the comparative information provided here will benefit future integrative approaches.

## Discussion

Maintenance of water status, a key process in plant functioning, is actively regulated on the whole plant scale (from root uptake to stomata) in response to variations in water availability. Given the known difference in drought sensitivity of the two genotypes, severe drought could yield two contrasting physiological states [32]. In our study, the physiological and transcriptional responses clearly indicate that both genotypes perceived water deficit as stressful. However, leaf water status was maintained and growth similarly hampered in both genotypes. The moderate stress levels applied induced similar physiological responses in both genotypes, allowing genotype-dependent transcriptional responses to be considered as intrinsic divergences in genome functioning rather than the result of the interaction between genome and physiological status. Drought sensing and metabolic adjustments involve tight molecular control [6]. We examined this control in poplar, analysing transcriptional remodelling in response to short and prolonged water deficits and in parallel in root apices and mature leaves. Given that poplar pathway information was inferred mainly from the arabidopsis literature, common gene names of the closest arabidopsis homolog were used to describe poplar genes (see Additional file 1 for correspondence) [38].

### Controlling energy and drought signalling under drought: a candidate process related to productivity

**Figure 5:**
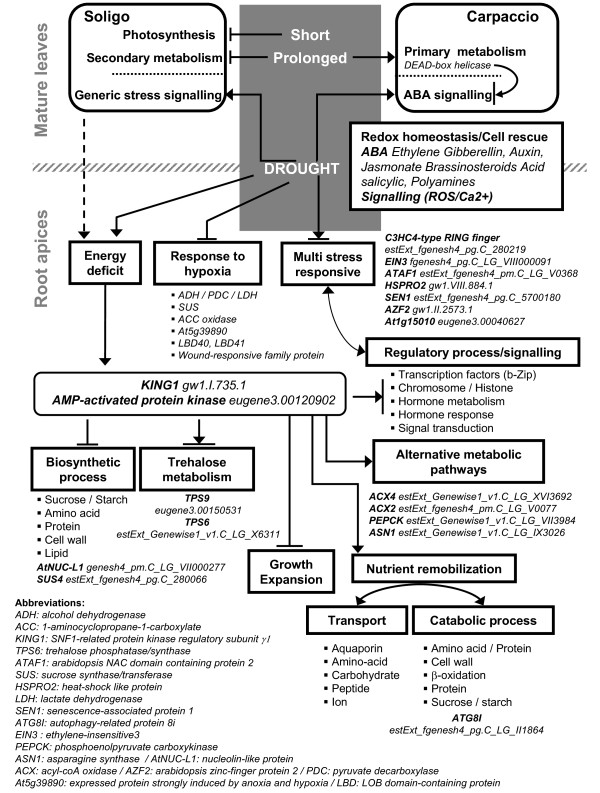
**Overview of drought-regulated transcriptome responses in mature leaves and root apices of two poplar genotypes**. Putative regulated processes are enclosed in boxes. For illustration, some representative genes are given in italic (The *Populus *genome v1.1). Gene regulation related to energy deficit response/signalling is described for arabidopsis [41,108].

Genes related to alternative metabolic pathways, transport and catabolism were up-regulated, and those associated to growth and biosynthesis were down-regulated by drought (Figure 5). This transcriptional remodelling suggested that an energy deficit could occur, especially in roots. To test this hypothesis, our results were compared with *KIN10*-targeted genes [39]. One-third of the 600 genes involved in energy signalling in arabidopsis were found to be drought-regulated in poplar (Additional file 6). The transcriptional remodelling, consistent with energy deficit signalling, was exacerbated in Soligo roots under short water deficit, and paralleled by the down-regulation of photosynthesis-related genes in mature leaves, which is consistent with a potential reduction of sugar transfer to roots. Our analysis indicates that this response could be mediated by a *KING *ortholog. Transcriptional remodelling regulated by SNF1-RELATED KINASES was not found in mature leaves, suggesting that trehalose-6-phosphate signalling could be inefficient in source organs, as previously described in arabidopsis [40]. This energy deficit transcriptional response is described here for the first time in poplar and need to be further validated. Energy saving processes in arabidopsis are believed to involve a reduction of expansion and growth [41]. The drought-induced reduction of Soligo productivity in the field might arise from its intrinsic sensitivity and responsiveness to energy deprivation. In parallel, stress responses were found to be more generic in Soligo than in Carpaccio. In leaves of the drought-tolerant genotype, some "*DEAD-box RNA helicases" *were up-regulated in response to LMI (Figure 5), concurrently to the down-regulation of *"Responsive to desiccation 22*" and putative "*MYC2*" (Additional file 6). In roots, most putative "*DEAD-box RNA helicases*" were down-regulated in accordance with maintenance of ABA signalling. These results suggest but not prove that the up-regulation of "*DEAD-box RNA helicases*" in leaves, by contributing to the transience of the stress response, could contribute to the drought tolerance of Carpaccio in the field [42].

### ABA biosynthesis and signalling under drought: consistent transcriptome responses in leaf and root

The up-regulation of *9-cis-epoxycarotenoid dioxygenase (NCED)*, *Phytoene synthase *and *β-carotene hydroxylase* (BETA-OHASE1) suggests enhanced ABA biosynthesis in roots under all drought conditions (Additional file 7). Consistently, this regulation has been previously associated with a peak in ABA content during poplar bud development [38]. BETA-OHASE could be involved in ABA biosynthesis and its over-expression enhanced stress tolerance in arabidopsis [43,44]. In leaves, transcript profiling suggested that ABA biosynthesis was no longer activated under prolonged drought--only *NCED *was up-regulated under short drought stress--although ABA signalling was still detected, suggesting long-distance transport of the phytohormone.

Concerning ABA-responsive genes, *Pyarabactin resistance-like (PYL) *were down-regulated and those of *Protein phosphatase type-2C (PP2C) *were up-regulated. *PYLs *encode ABA receptors that interact with PP2C as regulators of the ABA-mediated signalling pathway [45]. The opposite regulation of *PYL *and *PP2C*, either in roots or in both organs, was described in arabidopsis in response to ABA treatment [46]. As shown in Additional file 7, we detected several orthologs of *Dehydration-responsive-element binding protein *(*DREB2A*, *1A*, *1D *up regulated in roots and *DREB3 *repressed in leaves) and four predicted *ABA-RESPONSIVE-ELEMENT binding factors *known to be involved in signalling cascades [47]. Interestingly, a putative transcription factor, close to *Homeobox protein ATHB7 *and *ATHB12*, was up-regulated under all drought conditions. These ABA-induced growth mediators were up-regulated in response to water-deficit in arabidopsis [48]. Among up-regulated transcription factors were two putative *Responsive to desiccation 26--*a transcriptional activator in ABA signal transduction [49]. Eight orthologs of *Arabidopsis nuclear factor-Y (NF-YA or NF-YB*) were drought-regulated in poplar [50]. In arabidopsis, drought-driven induction of NF-YA5 and of NF-YB1 was ABA-dependent or ABA-independent, respectively [51,52]. Here, putative *NF-YA *and *NF-YB *were both up-regulated in Carpaccio roots. However, only putative *NF-YA *transcripts accumulated in EAR-treated leaves, which is in line with ABA-dependent activation. The *ZFP *family, encoding C2H2-type zinc finger proteins, was found to be drought-responsive in poplar. Three genes similar to *Salt-tolerance zinc fingers *and one similar to *Arabidopsis zinc finger protein 2 *were regulated by drought in poplar, in accordance with their responsiveness to ABA and abiotic stress in arabidopsis [53]. In addition, three drought-responsive genes in poplar were similar to *ZAT12*, which is involved in ROS and abiotic stress signalling in arabidopsis [54]. Among them, *ZFP2 *was up-regulated in roots under all drought conditions, in line with its induction by abiotic stress in poplar [55]. Three putative *RARE-COLD-INDUCIBLE (RCI2) *were up-regulated in poplar. In arabidopsis, most *RCI2 *genes are induced by ABA and abiotic stress, and are potentially involved in the regulation of plasma membrane potential [56]. RCI2-A contributes to salt tolerance by preventing over-accumulation of K^+ ^and Na^+ ^[57]. The stress-responsive plasma membrane protein COR413-PM is potentially involved in signal transduction [58]. Two putative *COR413-PM *were induced in roots in response to drought. As highlighted in Figure 4, drought repressed several genes in poplar that are known to be up-regulated in response to hypoxia in arabidopsis and in poplar [59-61]. Such down-regulation has been reported previously in ABA and/or drought responses and could reflect antagonism between ABA and ethylene signalling [62-65]. Accordingly, poplar response to drought implied cross-talk between hormonal pathways (hormone metabolism or/and signalling, Figure 4, Additional file 1) in accordance with the literature [66,67].

### Multi-stress responsive genes in poplar

WRKY transcription factors constitute a large family of plant-specific regulators controlling senescence and responses to stress and ABA [66,68-70]. In poplar, we detected 31 drought-responsive *WRKY*. In arabidopsis, *AtWRKY-53*, *-54 *and *-70 *were found to be structurally related, *AtWRKY-18 *and *-40 *could interact each other, *AtWRKY-53 *and *-70 *exhibited partial overlapping functions, and *AtWRKY-70 *and *-54 *counteracted accumulation of salicylic acid [71]. In silico analysis of poplar WRKY-40 and -53 (eugene3.00061944 and grail3.0007034202) revealed an EAR-motif--a potential signature of transcriptional repressors [72]. Putative *AtWRKY-53*, *-40*, *-18, -54 *and *-70 *exhibited similar expression patterns, being up-regulated in roots under prolonged drought and in leaves in the early response, and/or being repressed in leaves under prolonged drought. Conserved clusters 5 and 6a (Figure 3a) gathered nine *WRKY *and some co-regulated genes such as *NIM1-interacting1 *and a putative *Pathogenesis-related protein1 *[73]. In poplar, *WRKY *and a putative *Plant natriuretic peptide (AtPNP-A) *were co-regulated by drought as already observed in arabidopsis [73]. AtPNP-A, an extracellular signalling molecule, could affect water and solute transport in response to stress [74].

Reactive oxygen species (ROS) have been associated with stress sensing/signalling, and have emerged as important general signals [75]. Although the drought applied here was mild-to-moderate in degree, we highlighted the activation of oxidative detoxification processes. In maize under mild water deficit, ABA accumulation triggered the generation of ROS, which up-regulated the antioxidant system [76]. *Glutathion-S-transferase*, *Superoxide dismutase*, *Lactoylglutathione lyase*, *Catalase*, *Glutathione peroxidase*, and *Ascorbate peroxidase *were up-regulated in roots under all conditions, and in leaves more strongly under prolonged stress. Drought also induced the up-regulation of several genes involved in oxidative stress tolerance/response in arabidopsis (Additional file 7), such as *Alternative oxidase *(AOX prevents mitochondrial ROS formation), *Temperature-induced lipocalin*, *AT1G68440*, *Senescence-associated protein1 *or *Senescence-associated gene21 *[77-81]. In poplar roots, drought positively regulated two putative *heat shock-like protein (HSPRO2)*, which, in arabidopsis, were involved in tolerance to oxidative stress and were drought-and ABA-responsive [23,82]. The *ChaC-like family protein*, which was repressed in response to oxidative stress, were strongly down-regulated in poplar leaves [79]. In accordance with ROS production and detoxification processes, we detected up-regulation of four *raffinose synthases *in roots, of a *stachyose synthase *in leaves and of three *galactinol synthases *(two in roots and one in leaves). An increase in galactinol, raffinose and stachyose content could improve osmoprotection and ROS scavenging [83]. The poplar drought response also implied the induction of genes related to cell rescue, including detoxification and chaperone-like activities (*peptidyl-prolyl-cis-trans-isomerase*, *heat shock protein*, *dnaJ*).

In poplar, predicted *lipid transfer proteins (LTP) *and *remorin (REM) *were induced strongly in roots, and *late embryogenesis proteins *(*LEA) *were up-regulated in both organs. Similar inductions in response to drought were detected in arabidopsis [84]. Non-specific LTPs could be calmodulin-binding, implying a possible Ca^2+^/CaM signalling function [85]. *AtREM4.1 *and *AtREM4.2 *were induced strongly in response to osmotic, salt, drought, ABA and brassinosteroid treatments [86]. Stress responsive LEAs are suspected to act as chaperones and/or ROS scavengers, to bind metal ions or divalent cations [87]. Among the four *Heat-stable protein1 *(*HSP1*) up-regulated in roots, eugene3.00101442 was specifically regulated in Carpaccio. In *Populus tremula*, SP1 was identified as a new stress responsive protein [88]. In poplar, we detected several genes that are commonly down-regulated by drought in arabidopsis [23]. Among them, two putative *Germin-like *(*AtGER1 *and *AtGER3*) were repressed under short water deficit but induced in response to LMO. AtGER1 and AtGER3 may be involved in the control of synthesis of cell wall polysaccharides and/or in scavenging of extracellular nucleotide-sugars [89,90]. In poplar, the two *ESKIMO *orthologs, namely gw1.VIII.1375.1 and gw1.X.1696.1, were repressed under most drought conditions. In arabidopsis, ESKIMO1, a positive regulator of transcription, negatively regulates cold acclimation [91]. The *eskimo1 *mutant was not drought-or salt-tolerant although *ATHB7*, *ATHB12 *or *PP2C *were constitutively up-regulated [91]. However, *Eskimo1 *is suspected to play a role in whole-plant water economy in arabidopsis [92]. While poplar gene functions are extrapolated mainly from sequence similarities, the transcriptome analysis performed here has given new insights into their involvement in cell physiology.

## Conclusions

Comparative genomics is a powerful tool that can help decipher the molecular basis of drought responses and reveal physiologically relevant processes. Reliable stress markers were extracted as well as genes whose expression differed in tolerant and sensitive genotypes. Similarly, analysis of variance detected a strong genotype effect in the transcriptome responses of poplar leaves to drought [14]. However, when using multi-species designed arrays, the risk of misinterpreting divergent signals has to be acknowledged and controlled for. We used a genomic DNA-based selection strategy to improve the detection of differentially expressed transcripts. Hybridizing genomic DNA was previously used in genotyping arabidopsis accessions and for analysing transcriptomes by cross-hybridization (banana/rice, chimpanzee/human) [93-95]. Our quantitative analysis of gene expression in poplar provides an unambiguous comparison of two hybrid transcriptomes. Although requiring to be tested further (wider range of organs, other poplar species, field conditions) our meta-analysis has revealed several candidate genes and processes that are differentially regulated in root and leaf, potentially under developmental control, and preferentially involved in rapid and long-term response to drought. Since most of these genes were not previously ascribed to poplar drought response, our work provides expression data that will enrich our knowledge of gene function in *Populus*.

## Methods

### Plant material

Cuttings of two *Populus deltoides *W. Bartram ex Marshall *x Populus nigra *L., namely 'Carpaccio' and 'Soligo', were planted in 2L-pots filled with a peat-sand mix (50/50 V/V) amended with 1 g L^-1 ^of CaMg(CO_3_)_2 _and 4 g L^-1 ^of fertilizer (Nutricote 711, Fertil; http://www.fertil.fr/) and were grown for two months in a greenhouse. In order to favour the development of a dense root system, the initial stem was cut at a few centimetres above its base. This "detopped cutting" was then transplanted into a 10L-pot filled with the same substrate. A new stem was allowed to grow for 10 weeks without water limitation. Trees were assigned randomly to 4 modalities of water supply. For controls (CTL), evaporative demand was compensated by 4 to 6 waterings to field capacity per day. For short-term water deficit (EAR), irrigation was withheld for 36 hours prior to harvest bringing soil relative extractable water (REW) into the range of 20-35%. For long-term drought, soil REW was controlled by water supply 4 times a day as detailed in [21]. Soil REW was maintained for 10 days either at 20-35% (LMI) or at 10-20% (LMO). Ambient conditions depended on outside weather but temperature was maintained in the range of 19-26°C, humidity varied between 55 and 85% (day/night) and PAR between 400 and 950 μmol m^-2 ^s^-1 ^(cloudy versus sunny days).

For each genotype x treatment combination, six trees were assigned to ecophysiological monitoring. Leaf predawn water potential, leaf relative water content, leaf full turgor osmotic pressure, height growth rate, and gas exchange were measured as previously described [21]. Instantaneous water use efficiency (WUE_i_) was calculated as the ratio of net CO_2 _assimilation rate to stomatal conductance for water vapour. Six other trees were devoted to molecular analyses. Controls and treated plants were harvested simultaneously following random sampling, between 11:00 am and 3:00 pm. Mature leaves and root apices of each tree were harvested in parallel. Two mature leaves were cut and immediately frozen in liquid nitrogen. In less than 30 seconds, about ten 1-cm-long apices were sampled in the whole root system, frozen immediately in liquid nitrogen and stored at -80°C.

### DNA and RNA extraction, RNA amplification and array hybridization

Total genomic DNA from leaves was extracted, fragmented, labelled and hybridized as described in [93] with the following modifications. Fifty μg of gDNA were partially digested with DNAse1 (Promega, http://www.promega.com/). DNAse1 was heat-inactivated with 2 μL inactivation buffer (10 min at 65°C). gDNA fragments were labelled by adding 200 U terminal deoxynucleotidyl transferase (90 min at 37°C) and hybridized for 20 h at 45°C.

Total RNA of each sample was extracted separately. Total RNA was extracted from 100 mg of leaves and 30 mg of roots with an Rneasy Plant Mini kit, using a DNAse1 treatment (Qiagen, http://www1.qiagen.com/). After checking integrity (2100 Bioanalyzer, Agilent, http://www.home.agilent.com/), RNA was quantified (RiboGreen RNA Quantification Reagent, http://www.promega.com/). Amplification and hybridization on Affymetrix GeneChip Poplar Genome Arrays were performed according to the manufacturer's protocol (Affymetrix, http://www.affymetrix.com/). Arrays were scanned with the GeneChip Scanner 3000-7G piloted by the GeneChip Operating Software (GCOS).

### Microarray analyses

Transcriptome analysis was conducted using 36 Affymetrix GeneChip Poplar Genome Arrays. Four arrays were devoted to genomic DNA hybridization (two technical replicates per genotypes). For expression arrays and for all conditions (2 genotypes × 4 treatments × 2 organs), the six trees were assigned randomly to two biological replicates. In each biological replicate, the same individuals were pooled for both organs and the three individuals contributed equally to the pool of total RNA. All raw and normalized data are available through both the CATdb database [AFFY_POPSEC_Nancy_Roots_poplar, AFFY_POPSEC_Nancy_Leaves_poplar and AFFY_genomic_Poplar, http://urgv.evry.inra.fr/CATdb] and the Gene Expression Omnibus repository at the NCBI [GSE17223, GSE17226, GSE17230 and GSE21334; http://www.ncbi.nlm.nih.gov/geo/]. The 16 arrays devoted to root samples, 16 arrays devoted to leaf samples and 4 arrays hybridized with genomic DNA were normalized separately with the gcrma algorithm available in the Bioconductor package [96,97]. To determine which genes were differentially expressed between two given conditions, we performed a two group t-test assuming equal variance between groups. To fit the assumption of equal variance of gene expression per group, genes displaying extreme variation (too small or too large) were excluded from the analysis. The raw *P-*values were adjusted by the Bonferroni method, which controls the Family Wise Error Rate [98]. A gene was declared differentially expressed if the Bonferroni *P-*value was below 0.05. SR-EAR vs SR-CTL comparison had a lower variance than the others (0.037 *versus *a mean variance of 0.052 ± 0.001). Seven genes were selected for RT-qPCR validation (Additional file 8). For each condition, four out of the six samples were chosen randomly for the RT-qPCR procedure (as described in [99]; with 500 ng RNA). Similar gene expression patterns were obtained with both methods (Additional file 9).

In the probeset selection procedure, the background level was set to 3.1 on the basis of the mean signal intensities of 62 reporter probesets (i.e. several controls that are not in the investigated poplar genomes; mean value 2.5 and mean maximum value 3.5). For a given array, any probeset with a signal intensity below this cut-off value was labelled "absent". In the expression arrays, when a probeset was labelled "present" in the two biological replicates of a condition, the targeted transcript was considered expressed. For genomic DNA, the analysis was less stringent. We considered that hybridization was possible when the probeset was labelled "present" in at least one of two technical replicates.

### GO enrichment and detection of differentially expressed gene groups

Probesets were assigned to gene model (poplar genome v1.1, http://genome.jgi-psf.org/poplar/poplar.home.html) using the batch query of NetAffx Analysis Center http://www.affymetrix.com/analysis/index.affx, poplar database query at JGI and similarity researches at NCBI http://www.ncbi.nlm.nih.gov[28]. Sequence alignments were performed using BLASTn (default parameters and a maximal *e-*value of 10^-5^) and only the best homologies were considered further. Following the release of phytozome v5.0 in January 2010, the best homology was confirmed using the annotation of the poplar genome v2.0 http://www.phytozome.net/poplar. We employed singular enrichment analysis (SEA, http://bioinfo.cau.edu.cn/agriGO) on up-regulated or down-regulated gene lists sorted for each condition [100]. SEA using plant GO slim was performed independently for each condition with the Populus Affymetrix Genome array as a background list, followed by correction for multiple testing. Functional annotation and Gene Ontology were retrieved by querying the annotation browser in agriGO with Affymetrix probesets, by querying the annotation batch function at PopGenie http://130.239.72.5/popgenie1 with the poplar gene model, and by querying the annotation tool at The Arabidopsis Information Resource (TAIR) http://www.arabidopsis.org [37,101]. Annotations from all origins were compiled to determine functional class enrichments using Iterative Group Analysis (iGA) [34]. The iGA procedure, which is based on hypergeometric statistic calculations, detects concerted changes in functional classes and assigns a probability of change (PC-values) to each functional class. For each condition, differentially expressed genes were sorted by their mean normalized expression ratio in ascending or descending order. The iGA procedure was applied separately for up-and down regulation to determine which functional groups are most enriched at the top of the sorted gene lists [102].

### Differential clustering algorithm

The DCA, first described by Ihmels *et al*., was performed using an R script http://www.R-project.org developed by Lelandais *et al*. [35,36]. Transcriptional modules in the reference genotype were detected using a hierarchical clustering algorithm (with 'hclust' function and with the 'ward' method for probeset agglomeration) and, for each module, the corresponding gene expression patterns in the target genotype were segmented into two different sub-clusters (labelled as "a" and "b") using the same hierarchical clustering algorithm. The DCA results are presented as a distance matrix between gene expression measurements (reference genotype in rows and target genotype in columns). Transcriptional modules of co-expressed genes were first defined in Carpaccio (reference genotype), and their corresponding probesets in Soligo (target genotype) were next clustered into two groups according to their expression measurements across Soligo arrays. Clusters were automatically assigned to four categories ("full", "partial", "split" or "no" conservation), calculating the mean correlation of probesets within and between sub-groups "a" and "b", and comparing the values obtained to a specific threshold T (T = 0.4 in this study).

### Meta-analysis

A set of unique gene models was delineated from the list of drought-responsive probesets. When gene annotation was multiple (cross-hybridization), data were discarded. Each gene was described by Affymetrix probeset identifiers, the *Populus *genome v1.1 gene name and the AGI code. For genes matching multiple probesets, the expression pattern was assessed by a "present call" in at least one probeset. Using Venn diagrams, our list was compared with previous poplar studies [11,14,17,18,21,37,61,103-106]. Impact of maturity level on gene expression under optimal conditions was assessed using exPlot and data referred as UMA-0030 in UPSC-BASE and detailed in [17,37,106,107].

## Authors' contributions

MBBT conceived the experimental design, advised by DLT and JPR; IH designed and conducted the data mining; DC, IH, MBBT, DLT, SB and NN performed the research; DC and ET performed annotation; GL contributed DCA analysis; MLMM developed statistical methods; SB, JPT, MLMM and JPR guided the Affymetrix procedures; DC, MBBT and IH wrote the manuscript. All authors read and approved the final manuscript.

## Supplementary Material

Additional file 1 **(Microsoft Excel file) List of significantly drought-regulated genes**. Annotation, Log 2 ratio values (treated vs respective control) and Bonferroni *P*-values are given for the 6,725 probesets displaying a significant change in signal intensity in response to drought in at least one pair comparison (no Log 2 ratio cut-off, t-test, Bonferroni *P *< 0.05). Red: up regulation, Green: down-regulation, Black: not significant; White: missing value. Click here for file

Additional file 2 **(pdf file) Functional category enrichment analysis (SEA) among differentially expressed genes**. For clarity, only the most enriched GO terms and their *P-*values are given for each pair comparison (treated vs respective control). SEA were performed independently for up and down regulated genes (no Log 2 ratio cut-off, *P *< 0.05). Click here for file

Additional file 3 **(Microsoft Excel file) Functional annotation enrichment analysis (iGA) among differentially expressed genes**. For each functional class, annotation, probability of change (PC), and number of changed versus total group numbers (%) are given as well as composition in genes (The *Populus *genome v1.1). Click here for file

Additional file 4 **(Microsoft Excel file) Gene lists of DCA clusters**. Distinct table is given for each DCA run. Table S4-A gives the 3,515 probesets that were significantly regulated at least twice across the twelve conditions (t-test, Bonferroni *P *< 0.05); Table S4-B gives the 652 probesets that were significantly regulated in mature leaves, at least twice across the six combinations; and Table S4-C gives the 2,410 probesets that were significantly regulated in root apices at least twice across the six combinations. DCA cluster assignation, gene annotation, Log 2 ratio values (treated vs respective control) and Bonferroni *P*-values are given. Red: up regulation, Green: down-regulation, Black: not significant; White: missing value. Click here for file

Additional file 5 **(Microsoft Excel file) Expression and regulation of drought-responsive genes in other *Populus *species**. For the set of unique gene models that was delineated from the list of drought-responsive probesets, the *Populus *gene names (v1.1), the annotations and the AGI codes are given as well as the gene expression and regulation patterns in our study and in the literature. Based on relative expression under optimal condition, each gene was assigned to subsets. L: leaf, R: root, X: not regulated, S: subset. Click here for file

Additional file 6 **(Microsoft Excel file) Comparison of poplar drought-responsive genes with the transcriptional program induced by KIN10, by starvation conditions and antagonized by sugar availability **[39]. The probesets identifiers, the *Populus *gene names (v1.1), the annotations and the AGI codes are given for all drought-responsive genes those orthologs were regulated in response to energy deficit in arabidopsis. Genes are gathered according to biological processes. Numbers of regulated genes per pair comparison are summed. Yellow: drought-regulation consistent with sugar feeding in arabidopsis: Blue: drought-regulation consistent with energy deficit in arabidopsis. Click here for file

Additional file 7 **(pdf file) ABA-mediated drought response in poplar**. Based on the literature and sequence homology with arabidopsis, putative ABA-related genes involved in drought response were identified. These genes were assigned to ABA biosynthesis (A, G), ABA-mediated signalling pathway (B, D), and response to ABA stimulus (C) as well as to cell rescue/detoxification process settled in response to ABA-mediated ROS production (H). Putative interactions with ABA-independent signalling pathway are shown (E). Genes (The *Populus *genome v1.1) and source are given in a table. Supporting literature [23,42-60,68,76-86,88,91,92,109-113]. Click here for file

Additional file 8 **(pdf file) Primer sequences used for RT-qPCR validation**. Click here for file

Additional file 9 **(pdf file) Validation of microarray results by RT-qPCR**. The Log 2 ratios were obtained either by RT-qPCR (a, b: -ΔΔCt) or by array analysis (c, d: intensity ratio). We compared the expression patterns of 4 selected genes in mature leaves (a, c) and of 5 selected genes in root apices (b, d). Gene models are given in Additional file 8. -ΔΔCt was calculated with *PP2A *as the housekeeping gene. Click here for file
