# Supplementary material for: Comparative transcriptomics of drought responses in Populus: a meta-analysis of genome-wide expression profiling in mature leaves and root apices across two genotypes
Source: BMC Genomics. 2010 Nov 12;11:630. doi: 10.1186/1471-2164-11-630 (PMC3091765; doi:10.1186/1471-2164-11-630)
Supplement: Additional file 2 — (pdf file) Functional category enrichment analysis (SEA) among differentially expressed genes. For clarity, only the most enriched GO terms and their P-values are given for each pair comparison (treated vs respective control). SEA were performed independently for up and down regulated genes (no Log 2 ratio cut-off, P < 0.05). [file 1471-2164-11-630-S2.PDF]

	Up-regulated terms		Down-regulated terms	
	Go term	*P*-value	Go term	*P*-value
**Cellular component**				
**CR-EAR vs CR-CTL**	thylakoid	3.1E-17	cell wall	1.6E-26
	nucleus	1.3E-12	external encapsulating structure	1.9E-24
	cell	2.6E-11	endomembrane system	2.9E-17
**SR-EAR vs SR-CTL**	cytosol	6.2E-27	endomembrane system	3.6E-69
	nucleus	2.4E-22	cell wall	5.0E-65
	cell	1.3E-15	cell	2.0E-64
**CR-LMI vs CR-CTL**	cell	6.7E-21	cell	2.1E-13
	endomembrane system	4.0E-15	cell wall	3.5E-11
	membrane	1.9E-12	cytosol	3.7E-10
**SR-LMI vs SR-CTL**	nucleus	4.1E-19	cell wall	1.8E-26
	cytosol	2.2E-16	external encapsulating structure	7.4E-25
	cell	2.8E-16	endomembrane system	1.3E-17
**CR-LMO vs CR-CTL**	cell	5.1E-39	cell wall	3.7E-13
	endomembrane system	1.7E-34	external encapsulating structure	9.0E-12
	membrane	1.0E-25	nucleus	6.4E-09
**SR-LMO vs SR-CTL**	cell	6.0E-17	cell wall	5.6E-27
	nucleus	3.2E-13	external encapsulating structure	2.2E-24
	endomembrane system	2.1E-12	endomembrane system	4.3E-17
**CL-EAR vs CL-CTL**	nucleus	8.40E-09	external encapsulating structure	4.00E-08
	endomembrane system	4.20E-08	cell wall	1.30E-07
**SL-EAR vs SL-CTL**	nucleus	2.10E-15	thylakoid	3.70E-16
	cell	2.20E-08	membrane	1.50E-06
**CL-LMI vs CL-CTL**	cell part	8.50E-10	cell wall	2.90E-13
	intracellular	4.30E-06	external encapsulating structure	4.50E-12
**SL-LMI vs SL-CTL**	endomembrane system	4.30E-04	membrane	7.60E-05
	vacuole	8.50E-04	vacuole	1.50E-04
**CL-LMO vs CL-CTL**	cell wall	2.60E-14	cell wall	1.60E-13
	external encapsulating structure	3.10E-14	external encapsulating structure	2.90E-12
**SL-LMO vs S-CTL**	vacuole	4.30E-05	cytosol	3.00E-09
	endomembrane system	5.40E-05	endomembrane system	3.90E-08
**Biological process**				
**CR-EAR vs CR-CTL**	response to stimulus	8.2E-87	response to stimulus	1.9E-26
	response to stress	5.0E-76	response to biotic stimulus	4.7E-26
	response to abiotic stimulus	3.2E-63	response to stress	3.8E-25
	response to endogenous stimulus	2.4E-50	multi-organism process	1.2E-23
**SR-EAR vs SR-CTL**	response to stimulus	1.5E-159	developmental process	1.2E-70
	response to stress	1.3E-122	multicellular organismal process	9.9E-70
	response to abiotic stimulus	7.3E-113	multicellular organismaldevelopment	1.4E-63
	response to endogenous stimulus	1.6E-99	growth	3.8E-61
	developmental process	4.7E-55	response to stimulus	1.6E-57
**CR-LMI vs CR-CTL**	response to stimulus	1.1E-83	response to stimulus	4.3E-38
	response to stress	4.1E-71	response to stress	7.3E-28
	response to abiotic stimulus	1.5E-69	developmental process	2.1E-27
	multicellular organismal process	5.5E-30	anatomical structure development	4.8E-25
	developmental process	1.3E-29	response to endogenous stimulus	5.8E-21

**SR-LMI vs SR-CTL**	response to stress	4.7E-82	response to stimulus	8.7E-56
	response to stimulus	5.9E-82	multi-organism process	6.7E-40
	response to abiotic stimulus	5.7E-69	response to biotic stimulus	6.3E-39
	cellular process	2.7E-33	response to stress	5.7E-32
	response to endogenous stimulus	9.6E-32	response to endogenous stimulus	1.6E-29
**CR-LMO vs CR-CTL**	response to stimulus	3.0E-120	response to stimulus	4.3E-46
	response to stress	2.9E-96	response to stress	7.2E-34
	response to abiotic stimulus	4.8E-77	response to biotic stimulus	3.0E-33
	response to biotic stimulus	4.7E-54	multi-organism process	5.8E-25
	multi-organism process	1.3E-51	response to endogenous stimulus	2.9E-22
	developmental process	3.5E-38	developmental process	1.0E-18
**SR-LMO vs SR-CTL**	response to stimulus	9.7E-145	response to stimulus	1.4E-67
	response to stress	2.7E-125	response to stress	1.2E-42
	response to abiotic stimulus	5.0E-108	response to biotic stimulus	6.6E-42
	response to endogenous stimulus	7.8E-59	multi-organism process	1.1E-39
	response to biotic stimulus	3.7E-48	response to endogenous stimulus	6.1E-26
	multi-organism process	1.4E-41	developmental process	2.0E-23
	developmental process	3.0E-35	anatomical structure development	5.2E-21
**CL-EAR vs CL-CTL**	response to stimulus	1.3E-63	response to stimulus	4.90E-10
	response to stress	2.8E-57	response to stress	5.40E-07
	response to abiotic stimulus	1.2E-45	response to endogenous stimulus	1.90E-06
	response to endogenous stimulus	5.7E-44	response to abiotic stimulus	9.80E-06
**SL-EAR vs SL-CTL**	response to stress	1.5E-50	response to stimulus	1.90E-12
	response to stimulus	5.1E-45	photosynthesis	5.60E-12
	multi-organism process	4.1E-37	response to endogenous stimulus	3.50E-09
	response to biotic stimulus	5.4E-37	response to stress	7.00E-08
**CL-LMI vs CL-CTL**	response to abiotic stimulus	3.10E-10	response to stimulus	2.30E-32
	response to stimulus	3.9E-09	response to biotic stimulus	2.20E-22
	response to stress	4.00E-09	response to stress	5.70E-19
	response to endogenous stimulus	3.20E-07	multi-organism process	3.00E-17
	primary metabolic process	3.50E-07	response to endogenous stimulus	4.80E-17
	metabolic process	4.70E-07	cell communication	2.90E-11
**SL-LMI vs SL-CTL**	response to stimulus	1.80E-17	secondary metabolic process	8.40E-22
	response to stress	7.10E-15	response to stimulus	4.00E-18
	response to abiotic stimulus	2.50E-12	metabolic process	2.20E-12
	response to endogenous stimulus	1.10E-11	biosynthetic process	5.90E-12
	response to biotic stimulus	6.50E-10	cellular biosynthetic process	6.90E-12
	homeostatic process	1.60E-09	response to biotic stimulus	1.6E-11
**CL-LMO vs CL-CTL**	response to endogenous stimulus	1.40E-16	response to stimulus	2.10E-32
	response to stimulus	2.20E-16	response to biotic stimulus	1.30E-26
	response to stress	2.40E-15	multi-organism process	3.50E-25
	response to abiotic stimulus	1.60E-14	response to stress	2.20E-23
**SL-LMO vs SL-CTL**	response to abiotic stimulus	8.80E-41	response to stimulus	4.90E-43
	response to stress	8.00E-40	response to biotic stimulus	1.40E-31
	response to stimulus	2.20E-38	multi-organism process	2.10E-28
	response to endogenous stimulus	1.80E-18	response to stress	1.20E-22
	developmental process	3.20E-16	secondary metabolic process	4.80E-16
**Molecular function**				
**CR-EAR vs CR-CTL**	transcription factor activity	1.8E-30	catalytic activity	1.1E-11
	transcription regulator activity	1.0E-29	protein binding	1.3E-08

	binding	9.1E-29	binding	7.2E-08
	oxygen binding	8.2E-24	enzyme regulator activity	1.2E-07
**SR_EAR vs SR-CTL**	binding	2.4E-64	binding	1.4E-58
	transcription factor activity	2.8E-51	protein binding	3.5E-47
	transcription regulator activity	8.3E-51	catalytic activity	3.6E-36
	catalytic activity	2.0E-33	nucleotide binding	2.1E-24
**CR-LMI vs CR-CTLR**	binding	2.0E-30	binding	3.4E-23
	oxygen binding	3.7E-27	catalytic activity	1.0E-18
	transcription regulator activity	2.3E-17	oxygen binding	3.4E-12
	transcription factor activity	3.6E-16	protein binding	1.8E-11
	transporter activity	1.8E-12	Nucleotide binding	4.7E-08
**SR-LMI vs SR-CTLR**	binding	9.3E-52	oxygen binding	2.7E-19
	transcription regulator activity	4.2E-26	catalytic activity	4.0E-15
	catalytic activity	1.8E-23	binding	1.6E-13
	protein binding	5.6E-22	protein binding	4.5E-10
**CR-LMO vs CR-CTL**	binding	7.3E-34	binding	2.3E-13
	transporter activity	7.6E-20	catalytic activity	3.9E-12
	protein binding	8.9E-20	protein binding	5.2E-10
	catalytic activity	1.6E-19	enzyme regulator activity	2.60E-07
	transcription factor activity	4.7E-18	oxygen binding	6.10E-07
**SR-LMO vs SR-CTL**	binding	7.6E-44	catalytic activity	1.5E-18
	transcription factor activity	7.1E-36	binding	5.6E-18
	transcription regulator activity	2.0E-33	oxygen binding	2.0E-11
**CL-EAR vs CL-CTL**	transcription factor activity	1.10E-16	transcription factor activity	1.50E-04
	transcription regulator activity	4.50E-16	DNA binding	4.00E-04
	binding	9.00E-16	transcription regulator activity	5.10E-04
	oxygen binding	6.50E-14	binding	9.90E-04
**SL-EAR vs SL-CTL**	binding	1.20E-34	binding	9.00E-11
	transcription factor activity	8.20E-32	transcription factor activity	2.60E-06
	DNA binding	3.70E-20	DNA binding	2.40E-04
	protein binding	6.80E-18	nucleic acid binding	5.90E-04
**CL-LMI vs CL-CTL**	binding	1.70E-08	binding	6.20E-20
	hydrolase activity	3.90E-06	oxygen binding	2.00E-18
	catalytic activity	4.80E-06	transcription factor activity	1.40E-09
	nucleic acid binding	8.10E-06	kinase activity	1.60E-09
	nucleoside-triphosphatase activity	1.70E-04	catalytic activity	2.20E-09
**SL-LMI vs SL-CTL**	protein binding	1.60E-04	catalytic activity	4.20E-11
	transporter activity	1.90E-04	binding	1.10E-07
	hydrolase activity	7.70E-04	transporter activity	9.30E-04
**CL-LMO vs CL-CTL**	binding	1.50E-08	binding	2.30E-23
	hydrolase activity	8.80E-07	kinase activity	1.20E-17
	catalytic activity	3.10E-06	oxygen binding	9.40E-16
	transcription regulator activity	3.20E-04	transferase activity, transferring phosphorus-containing groups	1.80E-14
	transcription factor activity	3.60E-04	protein binding	4.00E-12
**SL-LMO vs SL-CTL**	protein binding	4.60E-15	catalytic activity	3.40E-25
	binding	3.30E-11	binding	1.40E-17
	hydrolase activity, acting on ester bonds	2.40E-07	oxygen binding	1.90E-16
	transcription regulator activity	5.80E-07	transferase activity	4.90E-14
