# Supplementary material for: Comparative transcriptomics of drought responses in Populus: a meta-analysis of genome-wide expression profiling in mature leaves and root apices across two genotypes
Source: BMC Genomics. 2010 Nov 12;11:630. doi: 10.1186/1471-2164-11-630 (PMC3091765; doi:10.1186/1471-2164-11-630)
Supplement: Additional file 7 — (pdf file) ABA-mediated drought response in poplar. Based on the literature and sequence homology with arabidopsis, putative ABA-related genes involved in drought response were identified. These genes were assigned to ABA biosynthesis (A, G), ABA-mediated signalling pathway (B, D), and response to ABA stimulus (C) as well as to cell rescue/detoxification process settled in response to ABA-mediated ROS production (H). Putative interactions with ABA-independent signalling pathway are shown (E). Genes (The Populus genome v1.1) and source are given in a table. Supporting literature [23,42-60,68,76-86,88,91,92,109-113]. [file 1471-2164-11-630-S7.PDF]

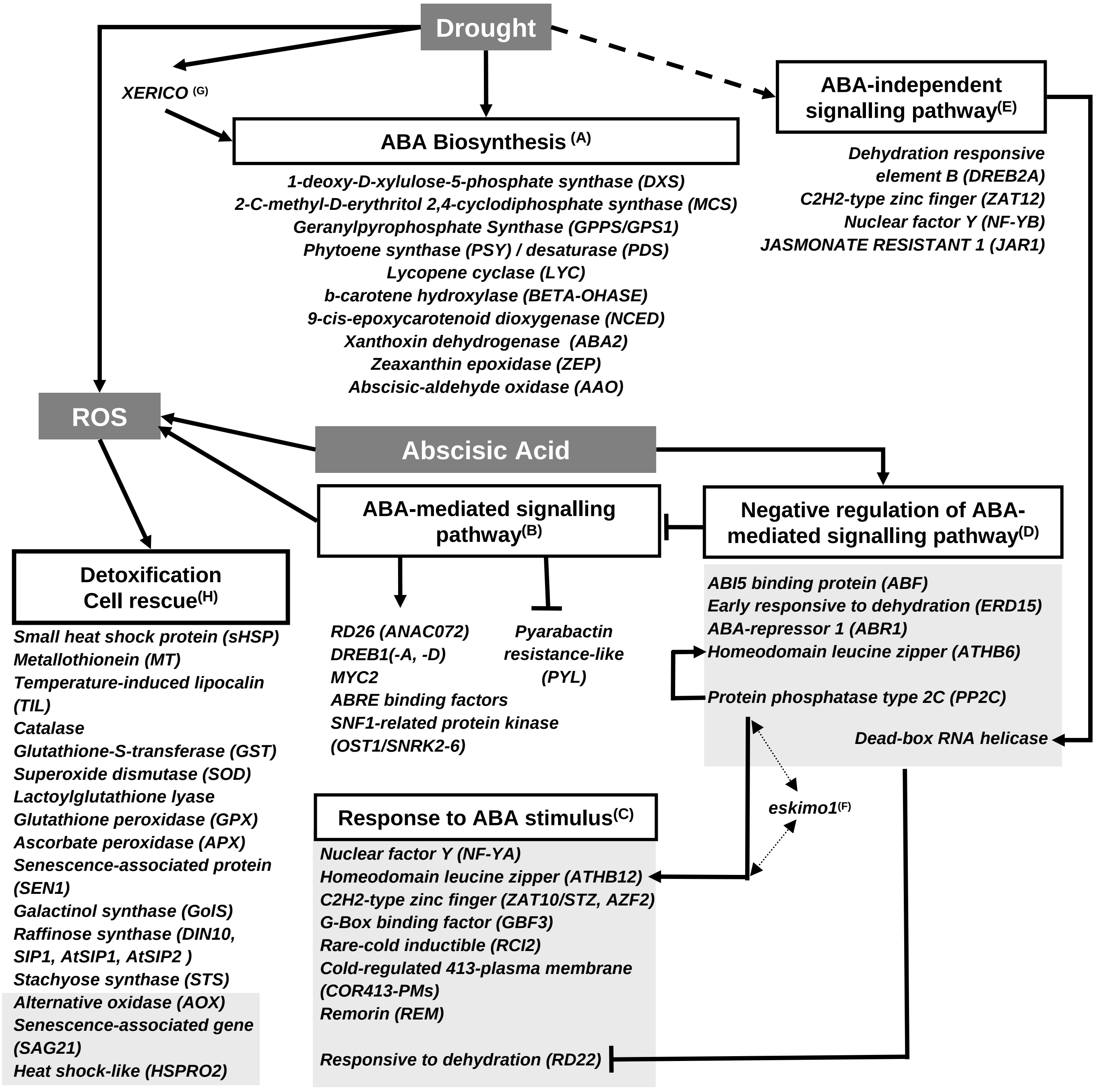

Drought
XERICO (G)
ABA Biosynthesis (A)
1-deoxy-D-xylulose-5-phosphate synthase (DXS)
2-C-methyl-D-erythritol 2,4-cyclodiphosphate synthase (MCS)
Geranylpyrophosphate Synthase (GPPS/GPS1)
Phytoene synthase (PSY) / desaturase (PDS)
Lycopene cyclase (LYC)
b-carotene hydroxylase (BETA-OHASE)
9-cis-epoxycarotenoid dioxygenase (NCED)
Xanthoxin dehydrogenase (ABA2)
Zeaxanthin epoxidase (ZEP)
Abscisic-aldehyde oxidase (AAO)
ABA-independent signalling pathway(E)
Dehydration responsive element B (DREB2A)
C2H2-type zinc finger (ZAT12)
Nuclear factor Y (NF-YB)
JASMONATE RESISTANT 1 (JAR1)
ROS
Abscisic Acid
ABA-mediated signalling pathway(B)
Negative regulation of ABA-mediated signalling pathway(D)
Detoxification Cell rescue(H)
Small heat shock protein (sHSP)
Metallothionein (MT)
Temperature-induced lipocalin (TIL)
Catalase
Glutathione-S-transferase (GST)
Superoxide dismutase (SOD)
Lactoylglutathione lyase
Glutathione peroxidase (GPX)
Ascorbate peroxidase (APX)
Senescence-associated protein (SEN1)
Galactinol synthase (GolS)
Raffinose synthase (DIN10, SIP1, AtSIP1, AtSIP2)
Stachyose synthase (STS)
Alternative oxidase (AOX)
Senescence-associated gene (SAG21)
Heat shock-like (HSPRO2)
RD26 (ANAC072)
DREB1(-A, -D)
MYC2
ABRE binding factors
SNF1-related protein kinase (OST1/SNRK2-6)
Pyarabactin resistance-like (PYL)
ABI5 binding protein (ABF)
Early responsive to dehydration (ERD15)
ABA-repressor 1 (ABR1)
Homeodomain leucine zipper (ATHB6)
Protein phosphatase type 2C (PP2C)
Dead-box RNA helicase
eskimo1(F)
Response to ABA stimulus(C)
Nuclear factor Y (NF-YA)
Homeodomain leucine zipper (ATHB12)
C2H2-type zinc finger (ZAT10/STZ, AZF2)
G-Box binding factor (GBF3)
Rare-cold inductible (RCI2)
Cold-regulated 413-plasma membrane (COR413-PMs)
Remorin (REM)
Responsive to dehydration (RD22)

Box	Gene symbol	The *Populus* genome v1.1
(A) ABA biosynthesis [43, 44, 109]	*DXS*	gw1.171.35.1 / gw1.III.2599.1
	MCS	gw1.I.8813.1*
	GPPS/GPS1	eugene3.00101320*
	PSD	fgenesh4_pm.C_LG_II000277*
	PSY	gw1.44.342.1*
	BETA-OHASE	estExt_fgenesh4_pg.C_440224* / estExt_Genewise1_v1.C_17880001
	LYC	grail3.0066009001
	NCED	eugene3.00110845* / gw1.146.74.1 / gw1.VI.2099.1
	ABA2	eugene3.00010285* / eugene3.00160678 / eugene3.00061405*
	ZEP	gw1.VII.2982.1* / eugene3.00160678
	AAO	eugene3.00102359
(B) ABA-mediated signalling pathway [45- 49, 51]	*RD26*	fgenesh4_pg.C_scaffold_107000083 / estExt_Genewise1_v1.C_LG_XI3994
	DREB1(-A,-D)	gw1.I.9165.1 / fgenesh4_pg.C_LG_XII001200
	MYC2	eugene3.00021640
	ABRE binding factors	eugene3.00021164 / grail3.0040023201* / fgenesh4_pg.C_LG_IX000628 / gw1.130.194.1 / grail3.0003013801*
	OST1/SNRK2-6	estExt_fgenesh4_pg.C_1300020*
	PYL	fgenesh4_pg.C_scaffold_118000009 / estExt_fgenesh4_pm.C_LG_III0464/ gw1.VI.730.1/ gw1.X.1600.1/ gw1.XVI.3905.1
(C) Response to ABA stimulus [48, 50, 51, 53, 55-60, 88]	*NF-YA*	grail3.0024038301
	ATHB12	eugene3.00140486*
	Zat10/STZ	estExt_Genewise1_v1.C_LG_XIV0633 / gw1.II.2573.1 / gw1.I.4086.1
	AZF2	eugene3.00090763
	GBF3	gw1.XIV.1557.1*
	RCI2	eugene3.00080380 / eugene3.01310022 / eugene3.00130015
	COR413-PMs	estExt_Genewise1_v1.C_LG_IV0366 / estExt_fgenesh4_pm.C_LG_IX0279
	REM	gw1.VI.1266.1 / gw1.XVI.1724.1
	RD22	gw1.801.1.1 / gw1.XIX.246.1
(D) Negative regulation of ABA-mediated signalling pathway [42, 46- 48, 51, 68, 111-113]	*ABF*	eugene3.00170499 / eugene3.00170500 / eugene3.00080900
	ERD15	estExt_fgenesh4_pg.C_LG_III1639
	ABR1	fgenesh4_pg.C_LG_V001123 / grail3.0003056501
	ATHB6	estExt_Genewise1_v1.C_LG_V4715 / gw1.VII.1686.1* / estExt_fgenesh4_pm.C_LG_II0461* / estExt_Genewise1_v1.C_LG_V2675*
	PP2C	gw1.XII.1331.1* / gw1.211.9.1* / fgenesh4_pm.C_LG_XII000477 / estExt_Genewise1_v1.C_LG_VIII0367* / grail3.0022022401* / fgenesh4_pg.C_LG_IX001205* / gw1.I.1127.1
	Dead-box RNA Helicase	estExt_fgenesh4_pg.C_LG_II1544 / estExt_Genewise1_v1.C_LG_XIV1670 / gw1.XIV.1672.1
(E) ABA-independent signalling pathway [47, 49, 52, 54, 56]	*DREB2A*	eugene3.00101772
	ZAT12	eugene3.00091367 / estExt_Genewise1_v1.C_LG_I1393
	NF-YB	gw1.XIV.2649.1
	JAR1	estExt_fgenesh4_pg.C_LG_II1531*
(F) [91, 92]	*ESKIMO1*	gw1.VIII.1375.1 / gw1.X.1696.1
(G) [110]	*XERICO*	estExt_Genewise1_v1.C_LG_XIV3857*
(H) Detoxification Cell rescue [23, 76 -86, 88]	*sHSP*	estExt_Genewise1_v1.C_LG_XIX2021 / grail3.0023024001 / grail3.0066002801 / eugene3.00080557 / estExt_Genewise1_v1.C_LG_IX3982 / eugene3.00011624 / gw1.I.502.1 / grail3.0022018701 / eugene3.00012051
	MT	eugene3.01070053 / eugene3.00110909 / eugene3.00060718 / gw1.I.1300.1 / eugene3.01200081 / eugene3.00060718 / estExt_fgenesh4_pg.C_2080003
	TIL	estExt_Genewise1_v1.C_LG_XVIII3
	Catalase	estExt_fgenesh4_pm.C_LG_V0695
	GST	eugene3.00020134 / gw1.X.2945.1 / gw1.VIII.2464.1 / estExt_fgenesh4_pg.C_8990003 / grail3.0094003001 / estExt_fgenesh4_pg.C_LG_VIII1530 / grail3.0036009801 / estExt_Genewise1_v1.C_860293 / gw1.942.4.1 / eugene3.00161127 / fgenesh4_pg.C_scaffold_3983000002 / gw1.7940.4.1
	SOD	gw1.XV.2559.1
	Lactoylglutathione lyase	estExt_Genewise1_v1.C_570289 / estExt_Genewise1_v1.C_LG_IV1582
	GPX	estExt_Genewise1_v1.C_LG_I8960
	APX	estExt_fgenesh4_pg.C_570071 / eugene3.00090344
	SEN1	estExt_fgenesh4_pg.C_570018
	GolS	estExt_fgenesh4_pm.C_LG_II0906 / fgenesh4_pm.C_LG_X000590 / gw1.131.196.1 / fgenesh4_pm.C_LG_VIII000417 / estExt_fgenesh4_pm.C_LG_XIII0025 / gw1.XIII.547.1
	DIN10	fgenesh4_pg.C_LG_VI000475 / grail3.4045000202
	SIP1	eugene3.00640058 / fgenesh4_pm.C_LG_VII000086
	AtSIP1	eugene3.01400033
	AtSIP2	estExt_Genewise1_v1.C_LG_XVI1715 / eugene3.00060481 / eugene3.00150654
	STS	eugene3.00140639
	AOX	gw1.XII.484.1
	SAG21	estExt_Genewise1_v1.C_LG_II1841
	HSPRO2	estExt_fgenesh4_pm.C_LG_X0749 / gw1.VIII.884.1

** : Gene models listed in ABA biosynthesis, ABA transcription factor or ABA signal transduction in poplar [38]*
[ ]: supporting literature
