# Supplementary material for: Comparative transcriptomics of drought responses in Populus: a meta-analysis of genome-wide expression profiling in mature leaves and root apices across two genotypes
Source: BMC Genomics. 2010 Nov 12;11:630. doi: 10.1186/1471-2164-11-630 (PMC3091765; doi:10.1186/1471-2164-11-630)
Supplement: Additional file 8 — (pdf file) Primer sequences used for RT-qPCR validation. [file 1471-2164-11-630-S8.PDF]

**Poplar gene model v1.1**<br>**Poplar gene model v2.0**	**Gene description**<br>**(Symbol)**	**Forward sequence**	**Reverse sequence**
gw1.XVI.3905.1<br>*POPTR_0016s13320.1*	*Pyarabactin resistance-like4 (PYL4)*	GTTGTTGGTGGGGACCATA	ACCGCGTAAGACTCCATGA
gw1.I.1127.1<br>*POPTR_0001s25200.1*	*Protein phosphatase type-2C (PP2C)*	ATGGTACACAGGAGATTTACC	GCCTAGGTGCTCGATTTAGA
estExt_fgenesh4_pg.C_LG_II0662<br>*POPTR_0002s07290.1*	*Alcohol dehydrogenase1 (ADH1)*	CGCATATACGGTCATGAAGC	GGTAAATTGGCTTTCCGTTG
eugene3.00140486<br>*POPTR_0014s09860.1*	*Homeobox-leucine zipper protein 7 (ATHB7)*	CGATGGCCTCTTCGATCAAT	TCACCAGGTGTGGAGTTCAT
estExt_fgenesh4_pm.C_LG_IX0624<br>*POPTR_0009s03230.1*	*Tonoplast intrinsic protein 1;3 (TIP1)*	GGGTGACATCGGGATAATTG	TGCAGAACCAACGAATGGAC
estExt_fgenesh4_pm.C_LG_IX0135<br>*POPTR_0009s14350.1*	*Expansin_like A2 (EXP)*	CATGGAGCAGTATGGGACAC	ATCGGTGATTTGGACTCCTG
eugene3.00061944<br>*POPTR_0006s27950.1*	*WRKY DNA-binding protein 40 (WRKY40)*	GTCCAGTTCCCAGTCCATCA	GAACTTCTGGCACTTGAAGGTTT
estExt_Genewise1_v1.C_LG_X0292<br>*POPTR_0010s13760.1*	*Protein phophatase 2A (PP2A)*	CATGTTTTGCTCCCACCTCT	CCGCAACTCTGTCTTCAACA
