# Supplementary figures and images for: Comparative transcriptomics of drought responses in Populus: a meta-analysis of genome-wide expression profiling in mature leaves and root apices across two genotypes
Source: BMC Genomics. 2010 Nov 12;11:630. doi: 10.1186/1471-2164-11-630 (PMC3091765; doi:10.1186/1471-2164-11-630)

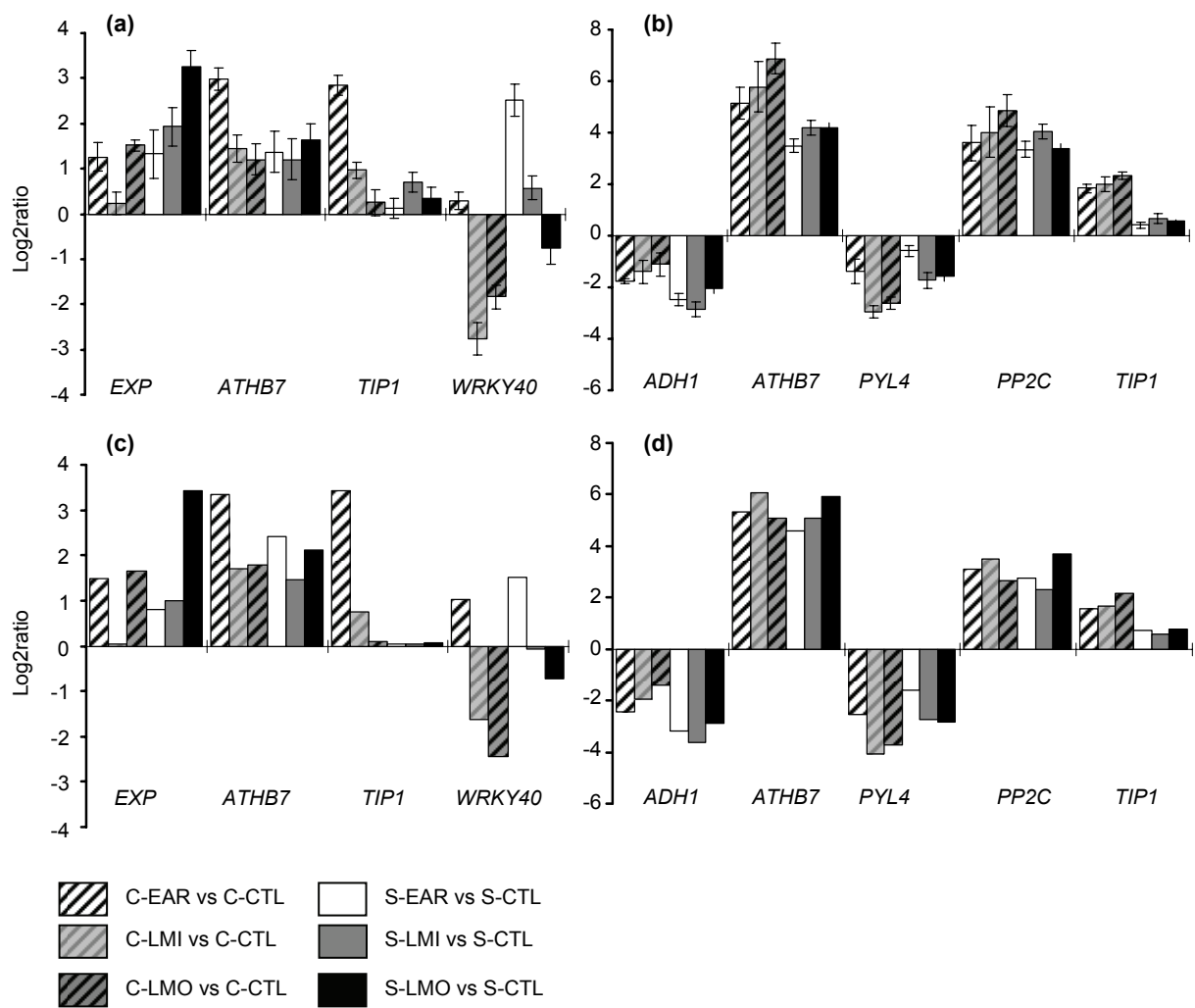

Supplement: Additional file 9 — (pdf file) Validation of microarray results by RT-qPCR. The Log 2 ratios were obtained either by RT-qPCR (a, b: -ΔΔCt) or by array analysis (c, d: intensity ratio). We compared the expression patterns of 4 selected genes in mature leaves (a, c) and of 5 selected genes in root apices (b, d). Gene models are given in Additional file 8. -ΔΔCt was calculated with PP2A as the housekeeping gene. [file 1471-2164-11-630-S9.PDF]
